# Recurrent laryngeal papillomatosis with pulmonary involvement: case report

**DOI:** 10.17843/rpmesp.2023.401.12169

**Published:** 2023-03-24

**Authors:** Enrique Zumaeta-Saavedra, Christian Chiara-Chilet, Julio Maquera-Afaray, Medalit Luna-Vilchez

**Affiliations:** 1 Universidad Continental, Lima, Peru Universidad Continental Lima Peru; 2 Instituto Nacional de Salud del Niño San Borja, Lima, Peru. Instituto Nacional de Salud del Niño San Borja Lima Peru; 3 Universidad Privada de Tacna, Tacna, Peru. Universidad Privada de Tacna Universidad Privada de Tacna Tacna Peru

**Keywords:** Human Papilloma Virus, Respiratory Papillomatosis, Microsurgery

## Abstract

Recurrent respiratory papillomatosis is a neoplastic disease caused by the human papillomavirus and characterized by the growth of exophytic proliferative lesions affecting the mucosa of the respiratory tract. This condition has a bimodal age distribution; the juvenile form affects those under 20 years of age, is more aggressive and presents multiple papillomatous lesions and high frequency of recurrence, compared to the adult form. Pulmonary involvement is rare and challenging to treat. We present the case of a 13-year-old male with a history of laryngeal papillomatosis since the age of two years. The patient showed respiratory distress and multiple stenosing nodules in the larynx and trachea, as well as several pulmonary cysts identified on chest CT. The patient underwent excision of the papillomatous lesions and tracheostomy. Then, the patient received a single dose of intravenous bevacizumab 400 mg and respiratory therapies with favorable evolution, without recurrences during follow-up.

## INTRODUCTION

Recurrent respiratory papillomatosis (RRP) also called recurrent laryngeal papillomatosis or glottic papillomatosis, is characterized by the growth of exophytic proliferative lesions of connective tissue covered by epithelium affecting the mucosa of the respiratory tract, caused by the human papillomavirus (HPV) ^1^^-^^3^. RRP is classified as high risk when it is caused by the HPV-16 and HPV-18 genotypes, which are associated with malignancy; and low risk when HPV-6 and HPV-11 genotypes are involved, which are associated with papilloma.

RRP exhibits a bimodal distribution, with a generally-aggressive juvenile form in those under 20 years of age, with multiple papillomatous lesions and high recurrence rates, compared to the adult form ^4^. Likewise, the frequency of the juvenile form of RRP is 4.3 cases per 100,000 population and 1.8 cases per 100,000 population in the adult form ^5^.

RRP is usually located in the larynx and subglottic region; involvement of the trachea and the lower airways has been documented in 5% and less than 1% of cases, respectively ^6^. Only 1.8% of patients presented pulmonary lesions during a 25-year follow-up study ^7^.

Surgical and medical approaches can be used to treat laryngeal papillomatosis. Surgical excision is the standard treatment ^8^. Bevacizumab is considered to be a safe alternative as adjuvant therapy, reducing the need for surgery, and having an impact on voice quality ^9^. In Peru, there are few reports of RRP ^10^^,^^11^. We present a case of RRP in a 13-year-old adolescent.

## CASE REPORT

A 13-year-old boy from Cusco, with a history of laryngeal papillomatosis since the age of two (he required tracheostomy at the age of three) and a mother with a history of genital papilloma. The patient was admitted to the Instituto Nacional de Salud del Niño de San Borja in Lima, with 16 days of illness, characterized by respiratory distress mostly at night, inspiratory laryngeal stridor and moderate dysphonia; he had previously received azithromycin and oxygen support, without improvement.

**Figure 1:**
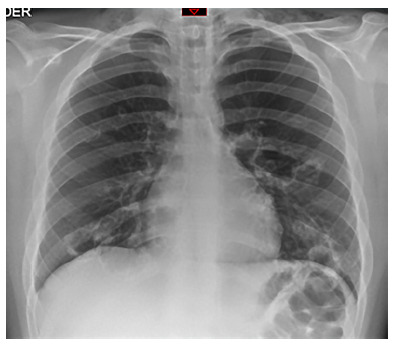
Chest X-ray. The image shows cervical subcutaneous emphysema, multiple cystic formations in both lungs, measuring approximately 38 x 26 mm as well as discrete pneumomediastinum.

**Figure 2:**
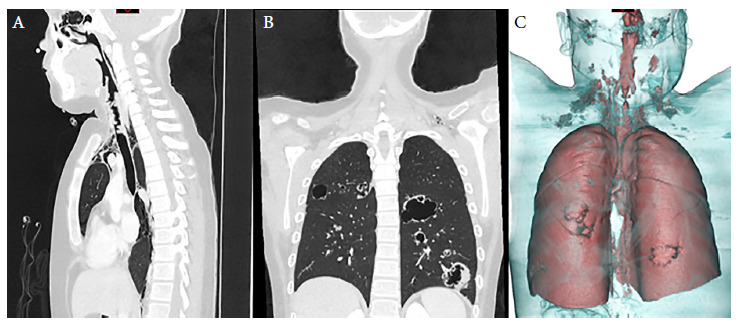
Head, neck and thorax tomography images. (A) Multiple nodules at the pharynx and trachea level can be seen stenosing the lumen. (B) and (C) cystic formations in both lungs, in addition to pneumomediastinum with emphysema of the subcutaneous tissue at the cervical level.

Physical examination showed mild subcostal retraction, decreased vesicular murmur in the left hemithorax and scarce wheezing predominantly in the right hemithorax. Therefore, he received oxygen support with a binasal cannula at 4 liters. The rest of the evaluation had no relevant findings. Laboratory tests showed leukocytes at 8.03 × 10^3^/u, platelets at 209 × 10^3^/u, hemoglobin at 13.2 g/dL, and C-reactive protein at 36.6 mg/L. Imaging tests included a chest X-ray (Figure 1) and a head and neck CT scan (Figure 2).

Forty-eight hours after hospital admission, the patient presented increased stridor and respiratory distress therefore he underwent a tracheostomy, microsurgery and excision of papillomatous lesions. An appendicular tumor with papillomatous appearance and ventricular bands in the glottic side of the epiglottis, vocal cords, subglottis and trachea up to ring 5 were found. The anatomopathological tests showed koilocytic atypia due to HPV and focal mild dysplasia.

Immediately after surgery, the patient was transferred to a pediatric intensive care unit for respiratory monitoring, and oxygen was weaned at 48 hours. He received a single dose of intravenous bevacizumab 400 mg and subsequently his condition improved. The patient remained hospitalized for seven days, after which he was clinically stable; oxygen saturation was normal and oxygen was weaned progressively. Then he was referred to Breña hospital for further management. The patient was evaluated by telemonitoring after eight months, and the family indicated that there was no evidence of relapse or other intercurrences.

## DISCUSSION

RRP, known as a benign neoplasm of the larynx, is caused by the HPV virus ^7^^,^^12^^-^^15^. The frequency of RRP is more than twice as high in the juvenile population compared to adults ^5^; moreover, young age is the most important factor for disease severity ^16^. Children diagnosed with RRP at a younger age (less than three years old) are 3.6 times more likely to have more than four surgeries per year and almost twice as likely to have multiple anatomical sites affected ^17^. The most common symptoms are hoarseness, chronic cough, dyspnea, recurrent upper respiratory infections, pneumonias, dysphagia, stridor or growth retardation, which tend to be more severe in children due to the rapid increase of the size of the lesions ^2^^,^^18^.

Currently, there is no cure for RRP and treatment is focused on maintaining permeable airways and voice quality; however, multiple surgical excisions are usually required in a short period of time, due to relapses ^16^^,^^19^. Several drugs such as interferon, cidofovir, bevacizumab, among others, have been suggested as adjuvant treatment ^16^. A review reported that cidofovir used as intralesional adjuvant therapy had a response rate of 56.6% against juvenile RRP; furthermore, the treatment did not increase the risk of laryngeal dysplasia ^21^.

Bevacizumab, also considered an alternative, is a recombinant monoclonal IgG1 antibody that binds extracellularly to the vascular endothelial growth factor (VEGF), which is an important mediator in the growth of lesions in RRP ^2^. This adjuvant therapy increases the time between surgeries, reduces the number of surgeries, and improves voice quality ^20^. Bevacizumab has been shown to be safe, even in patients with RRP requiring more than four surgeries per year^21^^,^^22^.

This report has the strength of presenting a case of RRP with pulmonary involvement confirmed by anatomopathological tests and tomography images with 3D reconstruction, as well as showing the positive effect of bevacizumab during treatment and follow-up. One of the limitations of this report is that the patient was referred to the Instituto Nacional de Salud del Niño Breña from Cusco right after surgery, therefore it was not possible to carry out face-to-face follow-up, endoscopic control and imaging in order to objectively evaluate the long-term effects of surgery and bevacizumab.

It is concluded that pulmonary involvement in RRP is unusual, with a higher prevalence in the younger population. This condition may have nonspecific clinical manifestations; therefore, imaging studies, endoscopy and biopsy should be used during diagnosis. In addition, this disease is characterized by recurrence, local complications and the possibility of becoming malignant, requiring surgery and adjuvant therapy with bevacizumab.
