# Supplementary material for: Recurrent laryngeal papillomatosis with pulmonary involvement: case report
Source: Rev Peru Med Exp Salud Publica. 2023 Mar 24;40(1):111–4. doi: 10.17843/rpmesp.2023.401.12169 (PMC10953631; doi:10.17843/rpmesp.2023.401.12169)
Supplement: Translation

# Translation: Papilomatosis laringea recurrente con compromiso pulmonar: reporte de caso

**Authors:** Enrique Zumaeta-Saavedra, Christian Chiara-Chilet, Julio Maquera-Afaray, Medalit Luna-Vilchez

**Affiliations:** 1 Universidad Continental, Lima, Perú.; 2 Instituto Nacional de Salud del Niño San Borja, Lima, Perú.; 3 Universidad Privada de Tacna, Tacna, Perú.

**Keywords:** Papiloma Virus Humano, Papilomatosis Respiratoria, Microcirugía

## Abstract

La papilomatosis respiratoria recurrente es una enfermedad neoplásica causada por el virus del papiloma humano y caracterizada por el crecimiento de lesiones proliferativas exofíticas que afectan la mucosa de las vías respiratorias. En su epidemiología se presenta una distribución bimodal, con una forma juvenil en menores de 20 años, más agresiva, con múltiples lesiones papilomatosas y alta frecuencia de recurrencia, en comparación con la forma adulta. El compromiso pulmonar es poco frecuente y su manejo es un desafío. Se presenta el caso de un varón de 13 años con antecedente de papilomatosis laríngea desde los dos años. El paciente mostró dificultad respiratoria y múltiples nódulos estenosantes en laringe y tráquea, y varios quistes pulmonares visualizados en la tomografía de tórax. Se le realizó exéresis de las lesiones papilomatosas y traqueostomía. Recibió dosis única de bevacizumab 400 mg intravenoso y terapias respiratorias con evolución favorable, sin recurrencias en el seguimiento.

## INTRODUCCIÓN

La papilomatosis respiratoria recurrente (PRR) también llamada papilomatosis laríngea recurrente o papilomatosis glótica, se caracteriza por el crecimiento de lesiones proliferativas exofíticas de tejido conectivo recubiertas por epitelio que afectan la mucosa de las vías respiratorias, originada por el virus del papiloma humano (VPH) ^1^^-^^3^. Se clasifica en alto riesgo cuando son causadas por los genotipos VPH-16 y VPH-18 asociados a malignidad y en bajo riesgo cuando los genotipos VPH-6 y VPH-11 asociados a papiloma son los causantes de la enfermedad.

La epidemiología de PRR muestra una distribución bimodal, con una forma juvenil presente en menores de 20 años, generalmente agresiva, con múltiples lesiones papilomatosas y alta frecuencia de recurrencia, en comparación con la forma adulta ^4^. Asimismo, la frecuencia de PRR es de 4,3 casos por 100 000 habitantes en la forma juvenil y 1,8 casos por 100 000 habitantes, en la forma adulta ^5^.

La PRR usualmente está localizada en la laringe y en la región subglótica, mientras que el compromiso de la tráquea se ha documentado en un 5% de los casos y las vías respiratorias inferiores están involucradas en menos del 1% de los casos ^6^. En un seguimiento a pacientes durante 25 años, solo el 1,8% de los pacientes presentaron lesiones pulmonares ^7^.

La papilomatosis laríngea tiene un tratamiento quirúrgico y médico, siendo el estándar la escisión quirúrgica ^8^. Como otra alternativa se usa el bevacizumab que es seguro y se emplea como terapia adyuvante, disminuyendo la necesidad de cirugías, con impacto en la calidad de la voz ^9^.

En el Perú, existen pocos reportes de PRR ^10^^,^^11^. Se presenta un caso de PRR en un adolescente de 13 años.

## REPORTE DEL CASO

Varón de 13 años procedente de Cusco, con antecedente de papilomatosis laríngea desde los dos años (a los tres años requirió colocación de traqueostomía) y madre con antecedente de papiloma genital. El paciente ingresó al Instituto Nacional de Salud del Niño de San Borja en Lima, por presentar un tiempo de enfermedad de 16 días, caracterizado por dificultad respiratoria a predominio nocturno, estridor laríngeo inspiratorio y moderada disfonía; previamente recibió azitromicina y soporte de oxígeno, sin mejoría. 

En el examen físico se encontró leve tiraje subcostal, murmullo vesicular disminuido en hemitórax izquierdo y sibilantes escasos a predominio del hemitórax derecho, por lo que requirió apoyo oxigenatorio con cánula binasal a 4 litros. El resto de la evaluación no tuvo hallazgos relevantes. A nivel laboratorial se evidenció leucocitos en 8,03 × 10^3^/u, plaquetas 209 × 10^3^/u, hemoglobina 13,2 g/dL, proteína C reactiva en 36,6 mg/L. Como parte de los estudios de imagen se le realizó una radiografía de tórax (Figura 1) y una tomografía de cabeza y cuello (Figura 2).

**Figura 1:** Radiografía de Tórax. En la imagen se observa enfisema subcutánea cervical, múltiples formaciones quísticas en ambos campos pulmonares, con medida de 38 x 26 mm aproximadamente; además discreto neumomediastino.

**Figura 2:** Imágenes de tomografía de cabeza, cuello y tórax. (A) Se aprecia a nivel de faringe y tráquea múltiples nódulos que estenosan la luz. (B) y (C) se observan formaciones quisticas en ambos campos pulmonares, además de neumomediastino con enfisema del tejido subcutáneo a nivel cervical.

A las 48 h de su ingreso presentó mayor estridor y dificultad respiratoria, por lo que se decidió su ingreso a sala de operaciones de emergencia para traqueostomía, microcirugía y exéresis de lesiones papilomatosas, se evidencio tumor apendicular de aspecto papilomatoso de bandas ventriculares en epiglotis cara glótica, cuerdas vocales, subglotis y tráquea hasta el anillo 5. El informe anatomopatológico reporto atipia coilocítica por VPH y displasia leve focal.

En el posoperatorio inmediato pasó a la unidad de cuidados intensivos pediátrica para monitoreo respiratorio, con destete de oxígeno a las 48 horas. Recibió dosis única de bevacizumab 400 mg intravenoso y posteriormente evolucionó con mejoría clínica. El paciente permaneció hospitalizado durante siete días, logrando la estabilidad clínica mediante la normalización en los niveles de saturación de oxígeno y el destete progresivo de oxígeno, fue posteriormente referido al hospital de Breña para continuar su manejo. Se realizó telemonitoreo luego de ocho meses y familiar indicó que no se ha evidenciado recaída u otras intercurrencias.

## DISCUSIÓN

La PRR, conocida como una neoplasia benigna de la laringe, es causada por el virus del VPH ^7^^,^^12^^-^^15^. La frecuencia de PRR es más del doble en la población juvenil comparado con los adultos ^5^; además la edad temprana es el factor más importante de gravedad de la enfermedad ^16^. Los niños cuyo PRR se diagnostica a una edad más temprana (menos de tres años) tienen 3,6 veces más probabilidades de tener más de cuatro cirugías por año y casi el doble de probabilidad de tener múltiples sitios anatómicos afectados ^17^. Los síntomas más frecuentes son ronquera, tos crónica, disnea, infecciones respiratorias superiores recurrentes, neumonías, disfagia, estridor o retraso del crecimiento, los mismos que tienden a ser más graves en los niños debido al rápido crecimiento de las lesiones ^2^^,^^18^.

En la actualidad, no existe cura para la enfermedad y el tratamiento se centra en mantener las vías respiratorias permeables y la calidad de voz; sin embargo, muchas veces se requieren múltiples escisiones quirúrgicas, en poco tiempo, debido a las recaídas ^16^^,^^19^. Dentro de la terapia adyuvante se ha sugerido el uso de múltiples medicamentos como interferón, cidofovir, bevacizumab, entre otros ^16^. En una revisión sobre el uso de cidofovir, adyuvante intralesional, se evidencio una respuesta de 56,5% de la PRR juvenil. Además, el tratamiento no aumentó el riesgo de displasia laríngea ^21^.

Otra alternativa es el bevacizumab, un anticuerpo IgG1 monoclonal recombinante que se une extracelularmente al factor de crecimiento endotelial vascular (VEGF), el cual es un mediador importante en el crecimiento de lesiones en la PRR ^2^. Esta terapia adyudante permite incrementar el tiempo entre cirugías, con una reducción en la frecuencia de estas y una mejor calidad de la voz ^20^. El medicamento ha demostrado ser seguro, incluso en pacientes con PRR que requieren más de cuatro cirugías al año ^21^^,^^22^.

El reporte tiene la fortaleza de presentar un caso de PRR con compromiso pulmonar documentado mediante estudio anatomopatológico e imágenes de tomografía con reconstrucción 3D, además de dar a conocer el efecto positivo del bevacizumab, posterior a su administración y seguimiento. Como limitaciones se debe reconocer que, debido a que el paciente provenía del Cusco y que luego de la intervención quirúrgica fue referido al Instituto Nacional de Salud del Niño Breña, no se pudo realizar un seguimiento presencial para la evaluación clínica, control endoscópico y de imágenes para corroborar de manera objetiva los efectos a largo plazo de la cirugía y del bevacizumab.

Se concluye, que el compromiso pulmonar en la PRR es una afectación poco frecuente, con mayor prevalencia en edades tempranas de la vida. Puede tener manifestaciones clínicas inespecíficas, por lo que el diagnóstico se debe realizar con estudios de imagen, endoscopia y biopsia. Además, esta enfermedad se caracteriza por su recurrencia, complicaciones locales y eventual malignización, siendo necesario un manejo quirúrgico y la terapia adyuvante con bevacizumab.
